# Chronic Alcohol Use Associated Encephalopathy With a Nearly Identical Presentation to Normal Pressure Hydrocephalus

**DOI:** 10.7759/cureus.38977

**Published:** 2023-05-13

**Authors:** Shreya Patel, Vasu Malhotra, Shani Scwartz, Travis Smith, Vikas Malhotra

**Affiliations:** 1 Medicine, Dr. Kiran C. Patel College of Osteopathic Medicine, Nova Southeastern University, Fort Lauderdale, USA; 2 Medicine, Dr. Kiran C. Patel College of Osteopathic Medicine, Nova Southeastern University, Clearwater, USA; 3 Internal Medicine, Lake Erie College of Osteopathic Medicine - Bradenton, Bradenton, USA; 4 Emergency Medicine, Lake Erie College of Osteopathic Medicine - Bradenton, Bradenton, USA; 5 Oncology, Florida Cancer Specialists, Fort Myers, USA; 6 Dr. Kiran C. Patel College of Osteopathic Medicine, Nova Southeastern University, Fort Lauderdale, USA

**Keywords:** wernicke-korsakoff syndrome, alcohol use encephalopathy, alcohol addiction, acute hydrocephalus, alcohol misuse, normal-pressure hydrocephalus

## Abstract

We present the case of a 52-year-old male who arrived at the Emergency Department after several ground-level falls in the past month. He complained of urinary incontinence, mild confusion, headaches, and appetite loss in the past month as well. Brain computed tomography (CT) and magnetic resonance imaging (MRI) were performed, which showed enlarged ventricles with moderately prominent cortical atrophy and no acute abnormalities. It was decided to conduct a cisternogram study with serial scans. The study showed a type IIIa cerebrospinal fluid (CSF) flow pattern at 24 hours. At the 48- and 72-hour marks, the study displayed an absence of radiotracer activity within the ventricles, while all the activity was concentrated within the cerebral cortices. These findings successfully ruled out normal pressure hydrocephalus (NPH) due to the highly specific indication of normal CSF circulation pattern. The patient was treated with thiamine and advised to quit drinking, as well as return for follow-up in one month as an outpatient for a repeat brain CT.

## Introduction

Hydrocephalus can be caused by many different etiologies, such as cerebral atrophy, normal pressure hydrocephalus (NPH), fibrosis, or obstruction in the ventricles. The most common etiology, however, is idiopathic. Hydrocephalus, by definition, is an accumulation of cerebrospinal fluid (CSF) in the ventricles. Chronic alcohol use can lead to impaired ependymal cilia mobility in the ventricles, disrupting the flow of CSF in the brain. Alcohol-associated encephalopathy, also known as Wernicke-Korsakoff syndrome, occurs in around 1-2% of the population in the United States [1]. It is most often seen in males, and patients of lower socioeconomic status. It is ultimately due to a thiamine (vitamin B1) deficiency, which occurs from excessive and chronic alcohol intake. Wernicke encephalopathy (WE), which usually presents first and is reversible, is diagnosed clinically with ocular dysfunction, ataxia, and confusion. Korsakoff syndrome (KS) presents later with confabulation and memory impairment. Appropriate labs to order would be liver function studies, thiamine levels, a complete blood count (CBC) for any macrocytic anemia present, and brain imaging such as a magnetic resonance imaging (MRI). Imaging can demonstrate cerebral or mamillary body atrophy, or hyperintensities in the thalamus and cerebellum. Treatment involves thiamine replacement and electrolyte repletion. NPH is a rare cause of hydrocephalus, and the idiopathic type is more commonly seen in the elderly population, with its incidence equal in men and women [1]. NPH is diagnosed using the clinical picture of urinary incontinence, ataxia, and dementia along with appropriate imaging. MRI is preferred and will demonstrate ventriculomegaly. It can also be diagnosed with a lumbar drain trial or cisternography. Treatment outcome varies with severity of presentation, using a ventriculoperitoneal shunt.

## Case presentation

A 52-year-old homeless male presented to the Emergency Department after a ground-level fall. He was unsure about how he fell but stated that he has been falling frequently, at least once per day, for the past few weeks. He denied loss of consciousness or hitting his head during the fall. He also had urinary incontinence, mild confusion, unsteadiness, mild headaches, and appetite loss for the past month. He denied chest pain, shortness of breath, palpitations, and vision changes.

**Figure 1:**
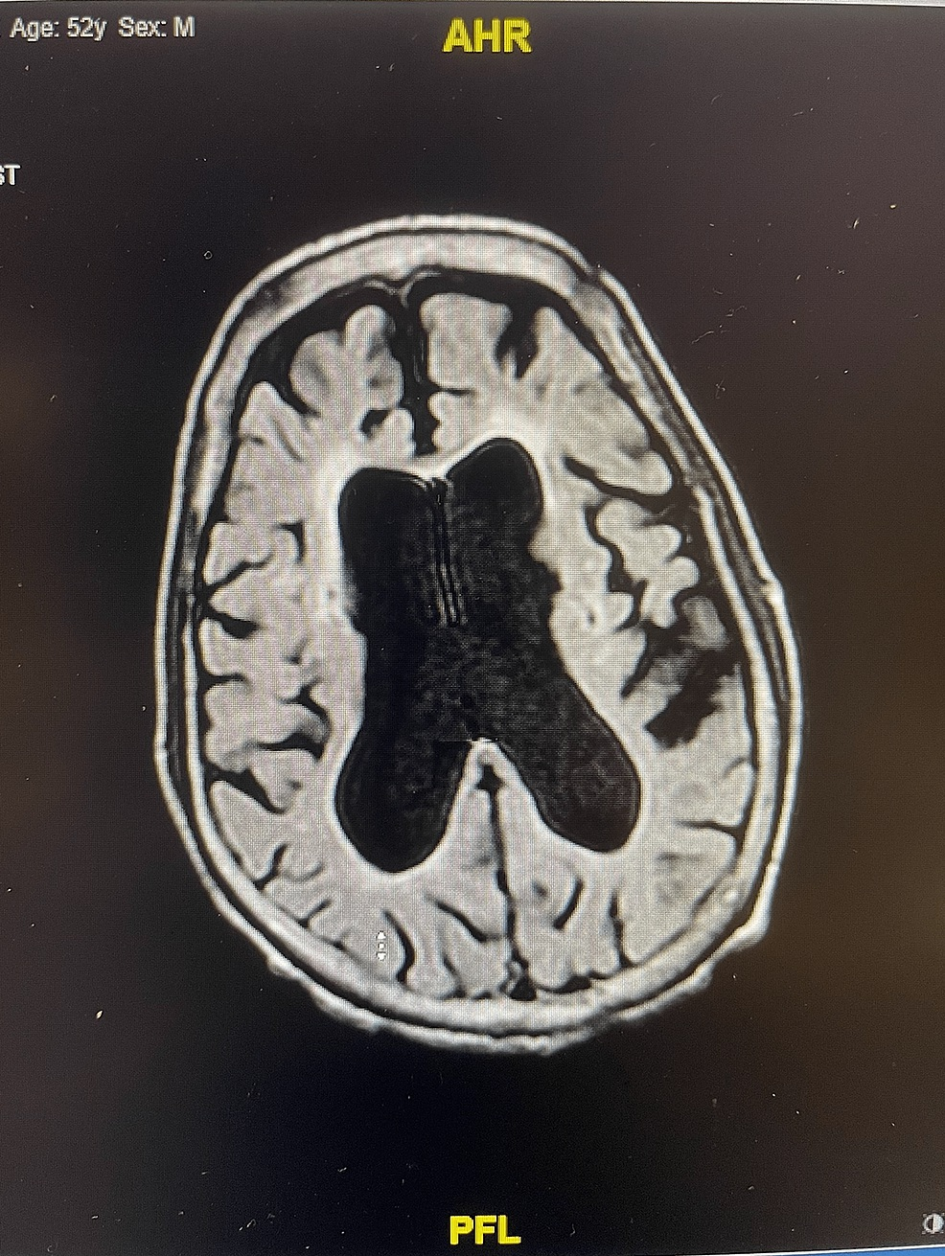
Brain MRI showing enlarged ventricles with moderately prominent cortical atrophy and no acute abnormalities. MRI, magnetic resonance imaging

**Figure 2:**
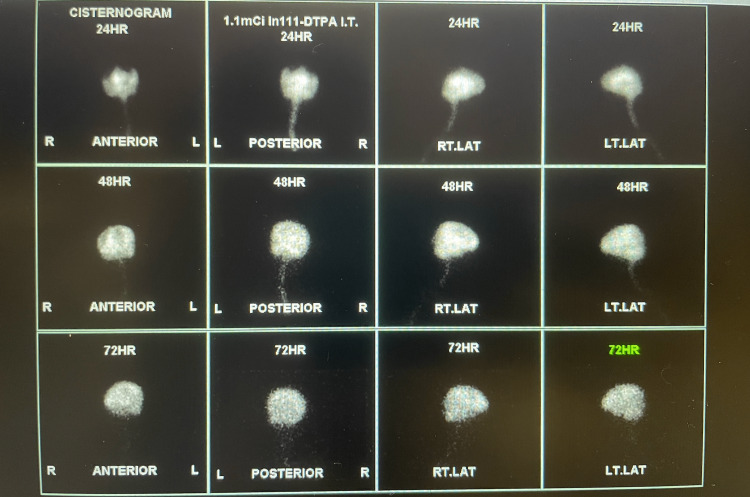
Type IIIa CSF flow pattern at 24 hours. At 48 and 72 hours, there it showed a lack of ventricular radiotracer activity with all the activity within the cerebral cortices. CSF, cerebrospinal fluid

He did not have a significant past medical history and had no home medications. He had a 10 pack-year history of smoking cigarettes, denied smoking marijuana, and had a history of four to five beers per day for many years. His vitals on admission to the Emergency Department were as follows: temperature of 36.4° C, heart rate of 77 beats per minute, respiratory rate of 14 breaths per minute, blood pressure of 159/89, and oxygen saturation of 97% on room air. Physical examination revealed a thin man, not in obvious distress lying in the hospital bed with notable temporal wasting. Significant lab values were as follows: hemoglobin of 9.2 g/dL (reference range: 13.5-17.5 g/dL for men) and platelet count of 652 x 10^3^/μL (reference range: 150-450 x 10^3^/μL). Urine drug screen was negative for opiates, methadone, barbiturates, amphetamine, cocaine, benzodiazepines, and marijuana. Brain computed tomography (CT) demonstrated no acute intracranial process and enlarged ventricles compared to the degree of sulcal enlargement. Alcohol level was 215 mg/dL (reference range for legal intoxication: 80 mg/dL or 0.08% blood alcohol concentration). Thiamine levels were unfortunately not available at presentation, which could limit the interpretation of this case. Brain MRI showed enlarged ventricles with moderately prominent cortical atrophy (Figure 1). Cisternogram (serial nuclear medicine brain scans) revealed the following: type IIIa CSF flow pattern at 24 hours, and at 48 and 72 hours, there was a lack of ventricular radiotracer activity with all the activity within the cerebral cortices (Figure 2). A lumbar puncture was performed, but the results were inconclusive, and the opening pressure was not recorded, which again may limit our understanding of the case.

The patient was diagnosed with chronic alcohol use associated encephalopathy due to the negative cisternogram results, ruling out NPH with a specificity close to 100%. He was treated with thiamine in the hospital and was then told to quit drinking alcohol and return for a repeat brain CT in one month. At the follow-up appointment, the repeat brain CT showed persistent ventricular enlargement and cortical atrophy, consistent with chronic alcohol use associated encephalopathy. Although these features are usually chronic and unlikely to reverse completely, the patient was counseled on the importance of abstaining from alcohol, continuing thiamine supplementation, and receiving appropriate medical care to manage his condition and prevent further deterioration. Given the patient's history of chronic alcohol use, other differential diagnoses such as alcohol-induced cerebellar degeneration and Marchiafava-Bignami disease were considered. However, the absence of specific signs and symptoms such as dysarthria, nystagmus, or corpus callosum lesions on imaging made these diagnoses less likely.

## Discussion

Hydrocephalus can be due to a wide variety of etiologies, one of which is excessive alcohol use [1]. Chronic alcohol use can lead to Korsakoff’s encephalopathy, which can cause permanent brain damage if not treated promptly. Additionally, NPH is a rare cause of hydrocephalus, with the idiopathic type usually seen in adults older than 60 years. Secondary causes of NPH can be from obstruction or fibrosis of the subarachnoid villi [1]. CSF is produced in the lateral ventricles by the choroid plexus. The direction of CSF flow is from the lateral ventricles to the third and fourth ventricles and then through the basal cisterns, tentorium, and subarachnoid space to the sagittal sinus. Decreased absorption of CSF leads to its accumulation in the ventricles, ultimately enlarging the ventricles on imaging [2]. Furthermore, excessive exposure to ethanol causes impaired ependymal cilia mobility, disrupting the normal flow of CSF through the ventricles and spinal cord [3]. This leads to a buildup of CSF in the brain, enlarging the ventricles and thus causing hydrocephalus. Alcohol-associated encephalopathy, or Wernicke-Korsakoff syndrome, occurs in around 1-2% of the population in the United States. This condition affects males more than females, and certain populations such as the homeless and psychiatric patients to a greater degree. Differences in the definition of NPH result in discrepancies in the incidence of this rare condition, varying from 2 to 20 million per year. Idiopathic NPH is more commonly seen in the elderly, while secondary NPH can be seen at any age. NPH is equally as common in males and females.

Chronic alcohol use is associated with a wide array of central nervous system disorders, including encephalopathy. Ventricular enlargement and cognitive dysfunction are commonly present in chronic alcoholics. Cerebral atrophy is also seen in chronic alcoholics, with a decrease in the volume of the white and gray matter of the brain. The classical triad of WE is ocular dysfunction, ataxia, and confusion. Korsakoff’s encephalopathy additionally presents with confabulation and memory impairment. NPH classically presents with the triad of urinary incontinence, gait abnormalities, and cognitive disturbance, and most commonly presents as gait apraxia, urinary incontinence, and dementia. The urinary incontinence can usually present as urge incontinence, and then as the cognitive impairment progresses, the incontinence gets worse.

The complications of chronic alcohol abuse are varied and multisystemic. One primary manifestation is KS, which describes a constellation of symptoms including, but not limited to, confabulation, personality changes, amnesia, and disorientation [4]. KS is typically preceded by an episode of WE, which is an acute and reversible presentation of similar symptoms from the same etiology. Both KS and WE are caused by a vitamin B1 (Thiamine) deficiency, as can be seen in chronic alcohol abuse due to malnutrition. The diagnosis of KS is largely based on clinical presentation; however, lab tests and imaging can aid in reaching a definitive diagnosis. Laboratory studies showing diminished vitamin B1 levels as well as diminished erythrocyte transketolase activity coefficient (ETKAC) activity can rule out other possible etiologies. Furthermore, elevated liver function tests (LFTs) and serum lactate and pyruvate levels can be seen in patients with KS. On MRI, the presence of mamillary body atrophy as well as hyperintensity in the thalamus and cerebellum are frequently present. All three symptoms of the triad (urinary incontinence, ataxia, and dementia) are not required to make a clinical diagnosis of NPH. However, gait abnormalities must be the predominant issue. A wide variety of diagnostic tests can be performed for patients with clinical NPH. MRI is preferred over CT to evaluate the size of the ventricles and sulci. Ventriculomegaly is defined by a modified Evans ratio (maximal diameter of the frontal horns of the lateral ventricles to the maximal width of the cranial cavity at the inner tables of the skull) greater than 0.3. Additionally, a confirmatory test is conducted due to the invasive nature of the treatment for NPH. Lumbar puncture or lumbar drain trial is performed to evaluate the CSF, and normal results and opening pressure are characteristic of NPH. Alternatively, a cisternography can be performed to visualize the distribution of the isotope through the cisterns, ventricles, and brain convexities. A cisternogram is an imaging study that uses radiolabeled isotype and takes serial scans at 1, 24, 48, and 72 hours. A diagnosis of NPH can be confirmed with the absence of isotopes in the brain convexities in the serial scans.

Treatment of chronic alcohol use disorder consists of thiamine replacement. Typically, high doses of IV thiamine, 500-1,500 mg, are given three times daily. Electrolytes and vitamins, in particular magnesium, are also replenished as they tend to be depleted as well in patients with alcohol use disorder. Regarding the NPH component, there is a very selective process in deciding which patient receives a ventriculoperitoneal shunt. Factors such as dementia, symptoms for over 6 months, advanced age, and presentation of urinary incontinence are associated with a worse surgical outcome.

## Conclusions

The purpose of this report is to highlight the importance of utilizing evidence-based medicine and radiology findings to correctly and accurately arrive to the best diagnosis and treatment for the patient. In this case, the clinical picture was leading more towards NPH with the history of excessive alcohol drinking, classic clinical triad of “wet, wobbly, wacky” and brain MRI revealing enlarged ventricles. The difference in treatment for chronic alcohol use associated encephalopathy versus normal pressure hydrocephalus is so distinctive that it is of the utmost importance for a clinician to correctly diagnose this condition. By using a highly specific and sensitive confirmatory test like the cisternogram, we were able to successfully rule out NPH and treat the patient accordingly.
